# Contribution of Cytokines to Tissue Damage During Human Respiratory Syncytial Virus Infection

**DOI:** 10.3389/fimmu.2019.00452

**Published:** 2019-03-18

**Authors:** Karen Bohmwald, Nicolás M. S. Gálvez, Gisela Canedo-Marroquín, Magdalena S. Pizarro-Ortega, Catalina Andrade-Parra, Felipe Gómez-Santander, Alexis M. Kalergis

**Affiliations:** ^1^Millenium Institute on Immunology and Immunotherapy, Departamento de Genética Molecular y Microbiología, Facultad de Ciencias Biológicas, Pontificia Universidad Católica de Chile, Santiago, Chile; ^2^Departamento de Endocrinología, Facultad de Medicina, Pontificia Universidad Católica de Chile, Santiago, Chile

**Keywords:** human respiratory syncytial virus, cytokines, chemokines, tissue damage, inflammation

## Abstract

The human respiratory syncytial virus (hRSV) remains one of the leading pathogens causing acute respiratory tract infections (ARTIs) in children younger than 2 years old, worldwide. Hospitalizations during the winter season due to hRSV-induced bronchiolitis and pneumonia increase every year. Despite this, there are no available vaccines to mitigate the health and economic burden caused by hRSV infection. The pathology caused by hRSV induces significant damage to the pulmonary epithelium, due to an excessive inflammatory response at the airways. Cytokines are considered essential players for the establishment and modulation of the immune and inflammatory responses, which can either be beneficial or harmful for the host. The deleterious effect observed upon hRSV infection is mainly due to tissue damage caused by immune cells recruited to the site of infection. This cellular recruitment takes place due to an altered profile of cytokines secreted by epithelial cells. As a result of inflammatory cell recruitment, the amounts of cytokines, such as IL-1, IL-6, IL-10, and CCL5 are further increased, while IL-10 and IFN-γ are decreased. However, additional studies are required to elicit the mediators directly associated with hRSV damage entirely. In addition to the detrimental induction of inflammatory mediators in the respiratory tract caused by hRSV, reports indicating alterations in the central nervous system (CNS) have been published. Indeed, elevated levels of IL-6, IL-8 (CXCL8), CCL2, and CCL4 have been reported in cerebrospinal fluid from patients with severe bronchiolitis and hRSV-associated encephalopathy. In this review article, we provide an in-depth analysis of the role of cytokines secreted upon hRSV infection and their potentially harmful contribution to tissue damage of the respiratory tract and the CNS.

## Introduction

### Prevalence of hRSV Infection Worldwide

**Figure 1:**
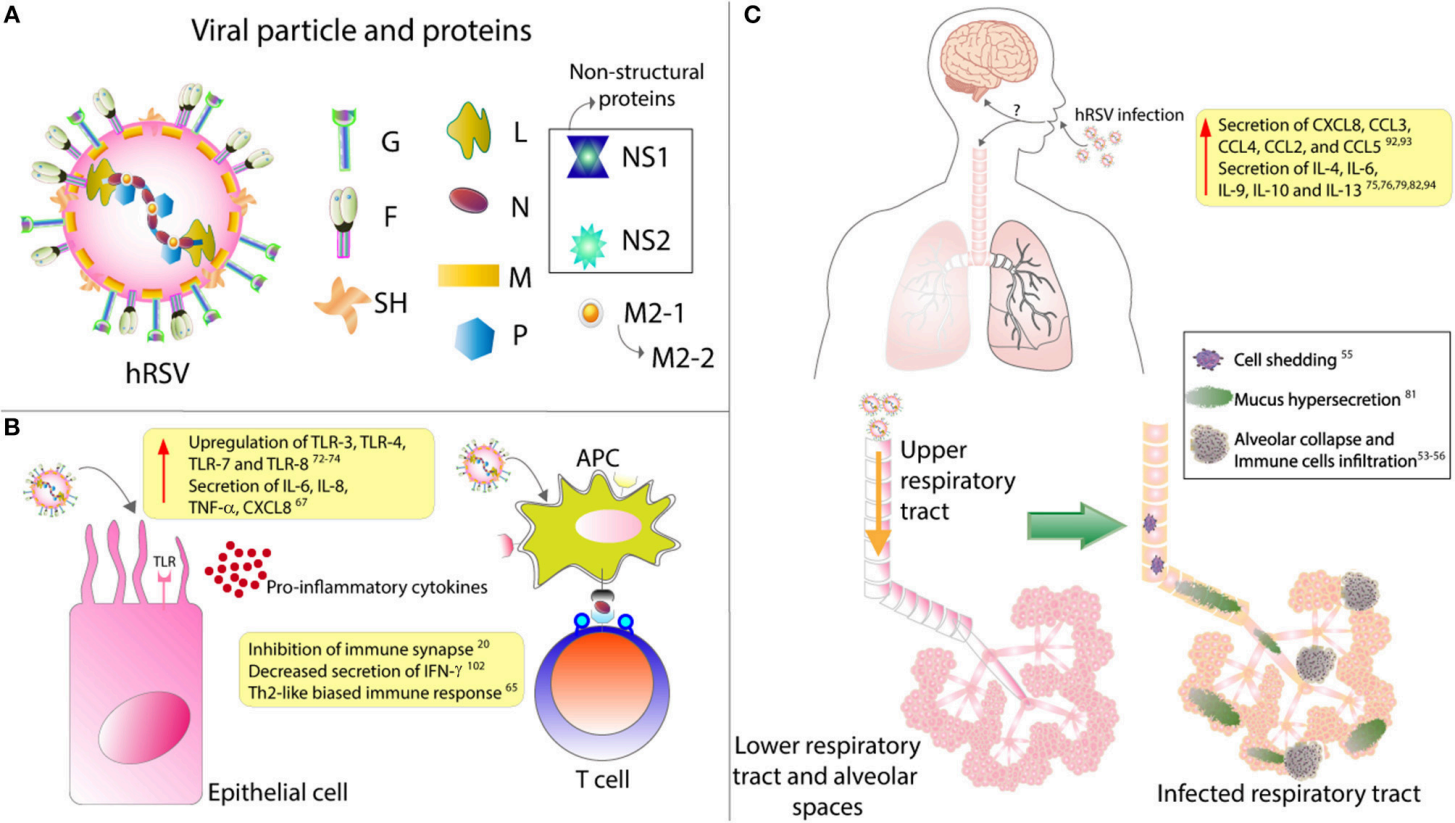
hRSV structure and impact over epithelial cells and respiratory tract. **(A)** The viral particle is composed of 9 structural proteins: 3 in the surface (F, G, and SH) and the other 5 inside the particle (L, N, P, M, M2.1, and M2.2). **(B)** Upon infection, it has been described that epithelial cells upregulates their expression of several TLRs and secrete mainly pro-inflammatory cytokines. Remarkably, it has also been described that hRSV infection of antigen presenting cells (APC) renders them unable to properly activate T cells, as this virus is able to inhibit the assembly of an immunological synapse. **(C)** hRSV infection induces the secretion of several pro-inflammatory cytokines that will induce the infiltration of many immune cells. These immune cells, along with the hypersecretion of mucus and the shedding of the infected epithelial cells, will induce the collapse of the lower respiratory tract. Finally, it has been described that hRSV infection can cause CNS pathologies, although the mechanism underlying this have not been described.

The human respiratory syncytial virus (hRSV) is one of the primary viral agents causing hospitalizations due to acute lower respiratory tract infection (ALRTI) in young children, immunocompromised and elderly individuals worldwide (1, 2). The epidemic period for hRSV infections usually takes place during the winter season in areas with temperate climates (3). This pathogen causes pulmonary manifestations mainly in the upper and lower respiratory tract, promoting the development of bronchiolitis and pneumonia (Figure 1) (4, 5). Some of the risk factors associated with the development of hRSV-associated ALRTI are premature birth, low birth weight, maternal smoking, history of atopy and no history of breastfeeding in infancy, among others (6). A recent report estimated that -during 2015- hRSV-associated ALRTI episodes reached a global burden of 33.1 million, resulting in 3.2 millions hospital admissions and around 60,000 in-hospital deaths in children under the age of 5 (1), although, no global studies of other populations such as the elderly or patients with underlying medical conditions have been conducted (7). Reinfections during childhood and adulthood are very common, and the severity of hRSV infections in healthy adults is mild. This decrease in severity has been related to higher neutralizing antibody titers induced by constant challenges with the virus throughout life (8). Besides children, the elderly have been described as another high-risk population, probably because of their senescent immune system (9). In this population, hRSV is the leading viral pathogen, which causes morbidity and mortality, followed by influenza A (10).

Besides the airway pathologies caused by hRSV, neurologic complications have also been described after infection with this virus, although less frequently (11–13). The etiology of the neurological alterations remains unknown. However, it has been proposed that inflammatory mediators, such as cytokines could be playing an essential role in the development of neurologic alterations (14, 15).

The hRSV is a highly contagious virus, as it can live outside of the host for about 6 h on hard surfaces, and as much as 20 min on the skin (16). Also, people that are infected with this virus remain contagious up to 8 days starting from the day of infection (17). Studies have shown that at least a third of the children experiencing hRSV infection within their first year of life will get re-infected during their second or third year of life (18). Patients infected with this virus cannot promote an adequate immunological response and, therefore, can get infected again with the same virus in the same cohort (19). In this regard, it has been described that this virus can impair the assembly of a proper immunological synapse between the antigen-presenting cells (APC), such as the dendritic cells, and T cells (Figure 1B) (20). In this way, hRSV renders T cells unable to respond correctly, which may lead to a poor adaptive immune response against the virus and, consequently, the reinfections mentioned above (Figure 1).

Most studies, aimed to determine the economic burden associated with hRSV, measure its immediate impact on health-care resources, such as hospitalizations, ambulatory care, and emergency department visits, focusing primarily on infant populations (21–23). It is noteworthy that hRSV has been associated with long-term illness such as asthma and recurrent wheezing (24, 25), which could represent a substantial increase in the economic burden related to this pathogen (26, 27).

Currently, there are no licensed vaccines available for preventing hRSV infection although several groups are working in the development of potentially effective vaccines and therapies. Nowadays, the only drug available on the market designed to ameliorate this disease is palivizumab, a humanized monoclonal antibody against the fusion protein (F-protein) of the virus. This product is used as a prophylactic option, along with ribavirin as a therapeutic option, although this strategy is only used in high-risk patients, such as children born after ≤ 29 weeks of gestation and preterm infants with chronic pulmonary disease (28, 29). Because this treatment fails to target most of the population susceptible to hRSV-caused disease, (i.e., healthy infants, children, and the elderly), the development of an effective vaccine is imperative (21, 22, 30). Several studies have concluded that the cost-effectiveness of palivizumab might not be enough to recommend the massive use of this antibody (22, 31–33). However, other studies have concluded that it does reduce the severity of infection and long-term effects on children, suggesting that it can diminish the spending of health-care resources (34, 35).

### hRSV: General Characteristics and Infective Cycle

The hRSV has been recently defined as a member of the *Orthopneumovirus* genus from the *Pneumoviridae* family being also recently renamed as human Orthopneumovirus and is an enveloped, negative-sense and single-stranded RNA virus with a genome of about 15.2 kb, possessing 10 genes that encode for 11 proteins (36–38). The viral particle displays 3 surface proteins, the F-protein, the glycoprotein (G) and the small hydrophobic (SH) protein (Figure 1A). Of all these, the G-protein is responsible for the attachment with the membrane of the host cell (39), mainly by binding to the CX3CR1 receptor on ciliated epithelial cells (40, 41). The fpre F-protein is responsible for the fusion of the viral membrane with the host cell membrane and further entry of the viral genetic material into the cytosol, apparently by its interaction with the surface protein nucleolin, although other receptors have been described to play a role in this process (42).

This virus can be transmitted by aerosol particles person-to-person, or via direct contact of these aerosol particles with the exposed mucosa, such as conjunctival (43). After infection, the incubation period can vary between 2 and 8 days in healthy individuals (44). At the beginning of hRSV infection, the virus meets the first line of defense of the organism, consisting of epithelial cells from the nasal and upper respiratory tract (45, 46).

The airway epithelium presents the apical junctional complex (AJC), which seals the space between the layer of epithelial cells and acts as a barrier that prevents the entry of pathogens into the organism (47). It has been described that hRSV infection induces a dysfunction in the epithelial barrier in a protein kinase D (PKD)-dependent manner (48). After infection by hRSV, cells exhibit a disruption of the AJC, which can be prevented when PKD-inhibitors are added, as described previously (48). As mentioned above, once hRSV reaches the apical side of the ciliated epithelial cells, the G and F proteins allow the attachment and fusion of the virus to the host membrane, respectively (39, 42).

After the virus has fused with the membrane of the host cells, it then begins the mechanism of entering the cells. The entry of the virus is through an endocytosis-dependent mechanism and allows the entering of the whole virus, including its lipid envelope (49). Then, the virus is carried within endocytic vacuoles, and undergoes a second fusion, this time with the vacuole itself, that occurs when the F-protein is cleaved by a furin-like convertase, to render the virus able to infect the cell (49). Then the virus reaches the cytoplasmic inclusion bodies (IBs) of the cells, where it can replicate its RNA using the viral RNA-dependent RNA polymerase (RdRp) complex, which is composed of the large protein (L) and the phosphoprotein (P) of the virus (50). As transcription goes on, the viral protein M2-1 is added to the complex allowing the synthesis of the mRNA (50, 51). The virus starts the replication of its RNA in the nasal epithelial cells and then it moves toward the bronchioles, where the replication becomes more effective (44). The virus spreads via intercellular extensions between two cells or through the cell to cell transmission, and in both cases, the infected cell is the one who passes the virus to the target cell (52).

To study the pathology associated with hRSV and the immune response during the infection, the use of several animal models has shown to be extremely important (53, 54). Lately, mice have been the animal model of choice for most immunology studies on this virus (53), although it is important to emphasize that the immune response observed in mice is not necessarily identical to the one observed in human patients. Some of these differences in the immune response between mice and humans are remarkable, for instance, the fact that older mice are more susceptible to hRSV-infection as compared to younger mice (55). Some techniques and methods to determine hRSV disease severity used in murine models are also different from those used to evaluate these parameters in humans. For instance, recording the body weight changes as a parameter of disease severity (more weight loss implies a more severe disease) is frequently used in the murine model, but it is not used as a parameter in humans disease (53). Also, obtaining bronchoalveolar lavage fluid (BALF) samples from mice is a standard procedure to evaluate inflammatory parameters, and these results can vary significantly from those observed in humans (53, 56). Although some differences can be observed, the data relative to cytokines and chemokines in the lower respiratory tract of mice and humans varies little, and to our knowledge, no published studies are describing these molecules in the upper respiratory tract and central nervous system (CNS) of mice (Table 1).

**Table 1:** Effect of hRSV infection on the expression profile of cytokines in the upper and lower respiratory tract and entral nervous system.

**Organism**	**Upper respiratory tract**	**Lower respiratory tract**	**Central nervous system**
Human		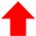 IL-6 (57, 58)	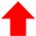 IL-6 (14, 15, 59)
		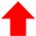 TNF-α (57, 58)	
		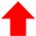 IL-4 (60–62)	
	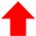 TNF-α (63, 64)	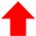 IL-6 (60)	
	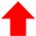 IL-12 (65)	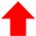 IL-9 (60, 66)	
	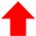 IL-23 (65)	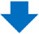 IL-10 (60–62, 67–69)	
		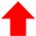 IL-13 (60–62)	
		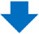 IFN-γ (66)	
		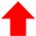 IL-17 (70, 71)	
		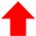 TSLP (72)	
		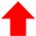 CXCL8 (57, 58, 73)	
	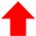 CXCL8 (74)	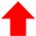 CCL3 (57, 58, 75)	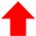 CCL2 (15)
	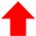 CCL5 (74)	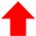 CCL4 (57, 58)	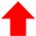 CCL4 (15)
	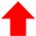 CXCL10 (74)	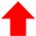 CCL2 (57, 58)	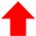 CXCL8 (15)
		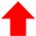 CCL5 (57, 58, 75)	
Mouse	–	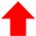 IL-6 (76)	–
		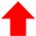 IL-1β (77)	
		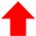 TNF-α (77)	
		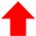 IFN-γ (77)	
		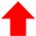 IL-12 (77)	
		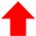 TSLP (78)	
	–	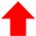 CCL3 (77)	–
		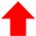 CCL5 (77)	

Further, in this review, we will provide an in-depth analysis of the current information available regarding the inflammatory mediators that are induced upon hRSV infection, and which ones are produced, up-regulated and down-regulated in the different sections of the respiratory tract and the CNS (Table 1). Additionally, we will discuss the contribution of cytokines to the immune response and immunopathology observed after hRSV infection.

## Cytokines Elicited by hRSV Infection

Among the inflammatory mediators that have been described to play an essential role in the hRSV pathology are cytokines and chemokines. Cytokines are small secreted molecules that contribute significantly to the modulation of the immune response and T cells differentiation (79). Several cell types can produce and secrete cytokines including immune cells, epithelial cells, and endothelial cells, amongst others (80, 81). Depending on the effect that they generate over immune cells, they can be classified into two groups; pro-inflammatory and anti-inflammatory (79). Interleukin (IL)-1, tumor necrosis factor alpha (TNF-α), interferon-gamma (IFN-γ), and IL-6, among others (79, 82, 83) belong to the pro-inflammatory group, IL-10 is anti-inflammatory, and IL-12 can be pro- and anti-inflammatory cytokine (Figure 1C) (84, 85).

Among cytokines, chemokines are a group of proteins with chemoattractant properties and are characterized by three to four cysteine residues present in their structure (80, 84). These proteins can be classified -according to the position of the cysteines residues in their N-terminal portion- into four families. The first family is the C-C chemokines present the cysteine residues continuously. The second family is the C-X-C chemokines present one amino acid between the two cysteine residues. The third family is the X-C chemokine only present one cysteine residue in a conserved position (this family is composed of only one member; XCL1). Finally, the four family is the C-X-3-C chemokine, which presents two cysteine residues separated by three interchangeable amino acids (This family possesses only one member; CX3CL1) (84, 86). Another relevant characteristic of chemokines is that they are considered to be promiscuous proteins, as they can interact with more than one chemokine receptor and one receptor can bind more than one chemokine (86). Additionally to their chemoattractant function, chemokines play an essential role in maintaining the homeostasis during the development of the brain, heart, and hematopoietic system, among others (86). Besides, they are also critical players in the modulation of the immune response during infections, as they are responsible for the infiltration of immune cells into the site of injury (84, 86).

## Cytokines Induced by hRSV Infection in the Upper Respiratory Tract

As mentioned above, the hRSV infection starts with the virus reaching the mucous membranes of the eyes, nose or mouth, allowing it to enter the organism (87). The first zone of infection is the upper respiratory tract, where it targets the ciliated epithelium of the nasopharynx, and then it moves toward the lungs, blocking the airways as the infection proceeds (Figure 1C) (88). This inflammation -known as bronchiolitis or pneumonia, accordingly to the degree of the disease- involves infiltration of polymorphonuclear cells (PMNs) such as neutrophils and eosinophils. Moreover, the rounding and shedding of the infected epithelial cells apparently caused by the NS2 protein, as described by Liesman et al. (74) inducing the collapse of the alveolar spaces and, therefore, impaired oxygen exchange (89). Remarkably, humans are born with at least a third of the alveoli that they will possess once the lungs are fully developed, with alveolar walls similar to the ones seen in an adult (90, 91). However, during childhood, these structures exhibit a lower area/volume ratio when compared with a fully developed lung, a rate that is increased until adolescence. Therefore, the useful space for gas exchange is reduced in early stages of human development. This phenomenon could explain for the exacerbated pathology observed in children, compared to teenagers and adults, with even further complications the younger they are (90, 91).

Several reports have described the changes in the ciliated epithelium upon infection with this virus. For instance, Wong et al. described that, upon infection, total loss of cilia is reported, mainly associated with microtubule damage (92). These could be in direct relation with reports indicating that this virus replicates in the apical cell surface of these cells (93). Remarkably, Smith et al. described that infection with hRSV could induce ciliary dyskinesia and ciliary loss of epithelial cells early during the infection, impairing in this way the clearance of the respiratory tract (94). Interestingly, Jumat et al. recently described the morphogenesis of hRSV in epithelial cells, using a primary culture of nasal epithelial cells as a model. They detected the presence of the F-protein of hRSV predominantly in cilia, but not the N-protein, observing this and several other proteins in the non-cilia locations of the cells, indicating that, probably the hRSV-F protein may be responsible for the damage to the cilia (46). Therefore, upon infection, hRSV seems to replicate and exit from non-cilia locations in the apical side of epithelial cells, somehow causing loss of ciliary function.

In response to all this damage, the airway epithelium generates cytokines and chemokines to recruit effector cells to the site of infection and restrict its propagation (95), causing an exacerbated immune response where infiltrating immune cells such as PMNs, T cells and inflammatory mediators cause damage to the tissues (63, 84). This exaggerated inflammatory response is increased as the infection progresses, with hRSV inducing a Th2-like immune response, promoting the inflammation (64). Notably, it has been described that primary infection with hRSV induces the transcription of nuclear factor kappa B (NF-κB) mainly through its M2-1 protein (96). This factor, in turn, produces the secretion of IL-8/CXCL8, TNF-α, CCL5, and CXCL10, among others. Accordingly, transcription factor AP-1 is also required for the expression of IL-8, as described by Dey et al. (97). Both NF-κB and AP-1 are regulated in their expression by the TGF-ß activation kinase 1 (TAK1), as deletion or inactivation of this kinase reduce gene expression of the transcription factors and decrease their nuclear translocation and DNA-binding activity (97), suggesting that the virus could be modulating these pathways. Finally, it has also been reported that STAT1 regulates the secretion of IL-4 by basophils upon infection with hRSV. In this line, Moore et al. described that KO mice for STAT1 showed higher levels of IL-4 in lungs, upon infection; a phenomenon that was reverted when mice were depleted from basophils. Remarkably, this increase in the expression of IL-4 correlated with more marked lung histopathology (98).

**Figure 2:**
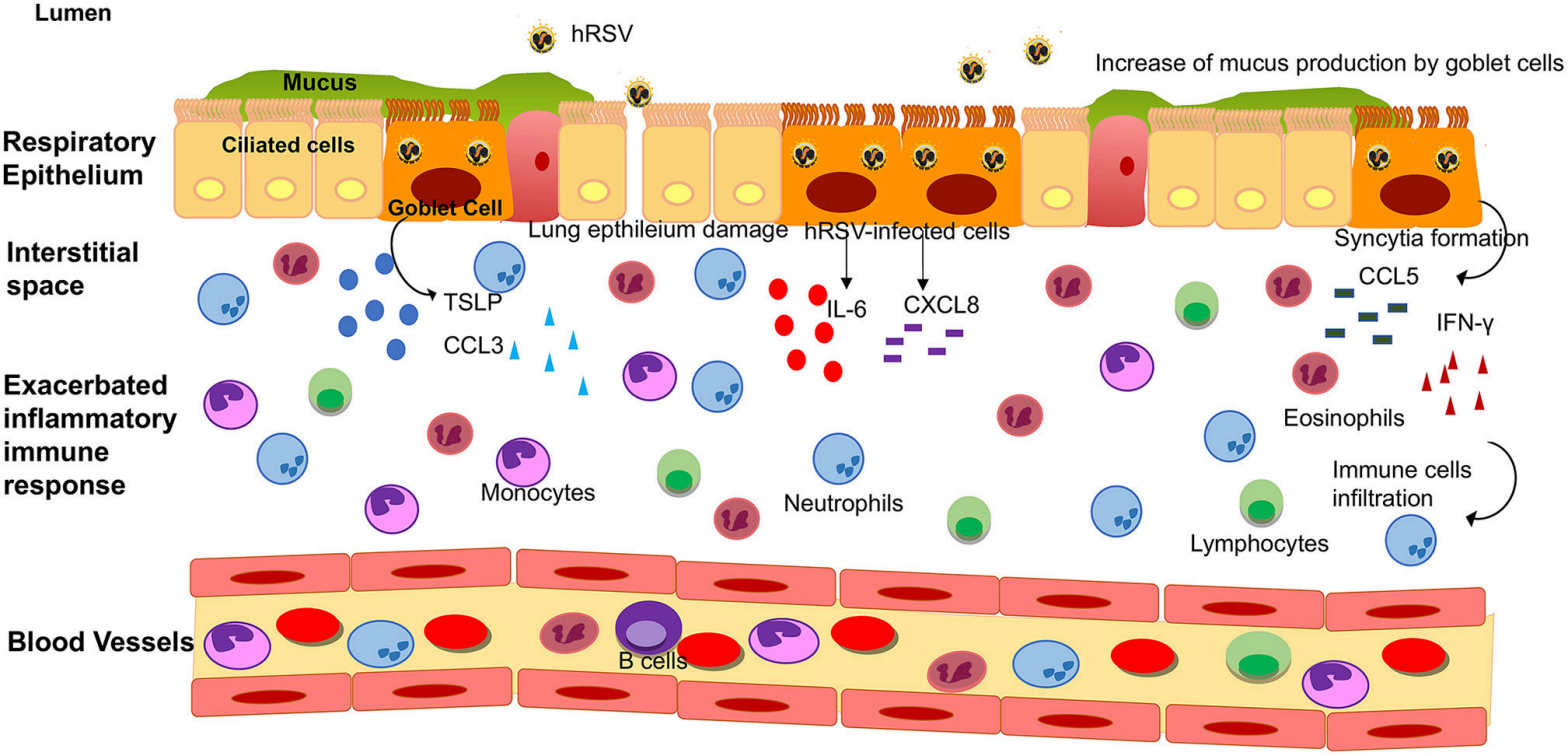
Lower respiratory tract inflammatory response induced by hRSV infection. Upon hRSV infection in the lower respiratory tract, the epithelial cells secrete IL-6, IFN-γ, CCL3, CCL5, CXCL8, and TSLP among others inflammatory mediators. This inflammatory immune response promotes the infiltration of immune cells (such as monocytes, eosinophil, neutrophils and lymphocytes, among other) into the lungs, causing an obstruction of the airways and damage to the tissue.

In light of all this, Das et al. reported that human nasal epithelial cells infected with hRSV exhibits increased levels of IL-6, CXCL8, and CCL5, as compared to non-infected cells (65). Remarkably, IL-2 levels in nasopharyngeal aspirates do not seem to correlate with hRSV infection, as Giugno et al. described that the concentration of this cytokine was heterogeneous among infected and non-infected children (99). The secretion of these pro-inflammatory cytokines may be adding to the exacerbated inflammation described in this disease (Figures 1C, 2).

Since hRSV-infected children are only brought onto health centers once the disease has reached an advanced development stage, it is hard to determine the temporality of the secretion of cytokines and chemokines in humans, during this disease. In this line, Blanco *et al*. performed a study in cotton rats where they measured the transcription levels of several of these molecules during primary and secondary infection (100). Therein the authors show an increase in the transcription levels of all the cytokines measured except for IL-10 during the first day post-infection. A peak for IL-6, IFN-α, and TNF-α, was detected during day 1 post-primary infection, decreasing the first two by day 3, while the latter remained high up until day 10. Likewise, IL-1β, CCL5, CXCL1, and CXCL10 transcription levels peaked at day 2, remaining high up until days 5 or 6. The cytokines that peaked during day 3 were IL-10 and CCL4, recovering normal levels by day 7. The last molecule to reach its peak was IFN-γ, at day 4 post-primary infection which correlates with previous studies indicating that this virus inhibits the expression of this cytokine. Then, at day 14, the levels of IFN-γ were returned to normal levels (100). Remarkably, infectious virus was not detected in the lungs of cotton rats challenged in a secondary infection; however, changes in the lung structure were detected even earlier than in primarily infected cotton rats (100). Despite all this, and as indicated above, these data are all related to transcript expression level, and it is not a direct measure of proteins. Therefore, this information must be taken into account cautiously.

Another work performed by Legg et al. examined the cytokine response to the hRSV through nasal lavage fluid in infants (101). In this study when some respiratory symptoms the research team visited the infant to whom they performed a clinical examination and nasal lavage, considering this collection of samples days 1 and 2. The same procedure was performed at day 5 and 6 since the development of the symptoms. They found that IL-4/IFN-γ ratio was elevated at day 1–2 and 5–6, and during the first two days, the IL-10/IL-12 ratio reached its peak (101). The results obtained with IFN-γ in infants correlates with the results obtained in mice since during the first couple of days the secretion of this cytokine has a similar pattern, suggesting that the other cytokines should behave similarly in humans as it does in cotton rats.

Toll-like receptors (TLRs) are pathogen recognition receptors (PRRs) that are activated upon the recognition of pathogen-associated molecular patterns (PAMPs) (102). They are expressed in several cell types such as immune cells and epithelial cells. Moreover, they are significant players in the early response against pathogens, as they can regulate the secretion of several cytokines and chemokines (103, 104). In this line, the role of TLRs in the innate immune response against hRSV is significant, as TLR3, TLR4, TLR7, and TLR8 are upregulated upon infection (Figure 1B) (105–107). TLR4 has been described to interact with the F-hRSV protein, leading to the activation of NF-κB and the secretion of the cytokines mentioned above, such as CXCL8 and TNF-α (108, 109). In humans, mutations in TLR4 impair the activation of this pathway and, in mice this renders the organism unable to clear the virus, and the persistence of the virus has been described in TLR4 deficient mice (57, 58). TLR3, which recognizes viral double-stranded RNA, induces the secretion of type I IFN and the activation of the NF-κB pathway (105). Remarkably, TLR3 deficient mice have shown a Th2-like biased immune response, further exacerbating the eosinophils infiltration and mucus secretion (60). TLR7, in turn, recognizes viral single-stranded RNA and induces the secretion of T cells-activator and mucus-secreting cytokines such as IL-12 and IL-23 (61).

Mucus secretion is also a significant factor associated with hRSV infection. The production of this thick layer that works as another defense mechanism of the organism is performed by goblet cells (Figures 1C, 2) (62). These cells are activated by cytokines such as IL-13, IL-17, and IL-23 (67), TLRs such as TLR3, and TLR7 (60) and immune receptors such as CXCR2 (68). As described above, TLR3 upregulation, secretion of IL-13 by infiltrating eosinophils and activation of several immune receptors are hallmarks of hRSV infection. Therefore, higher production of mucus usually correlates with more severe disease. Remarkably, Mukherjee et al. described that upon blockade of IL-17 through neutralizing antibodies, the secretion of mucus by hRSV-infected mice was significantly reduced, leading to a less exacerbated obstruction of the airways (69).

As it can be seen, the immune response against hRSV may be redundant at some points, but this redundancy itself is in part aiding the exacerbated inflammation and the production of pro-inflammatory cytokines. Although the organism exhibits several mechanisms to impede the advance of hRSV throughout the upper respiratory tract, this virus can avoid and even take advantage of many of these, eventually reaching the lower respiratory tract, where it will continue to replicate and progress in its pathology.

### Cytokines Induced by hRSV Infection in the Lower Respiratory Tract

It has been described that hRSV is a mucosa-restricted virus, as in natural infections it initially replicates in the epithelium of the nasopharynx (110). In immunologically naïve infants, hRSV spreads through a cell to cell transfer and extracellular binding, producing discontinuous foci of infection in the tracheal epithelium (110). The lower respiratory tract is essential for the respiratory system and is composed of trachea, bronchi (primary and secondary) and alveoli (111). Under normal conditions, inhaled pathogens are cleared via the mucociliary escalator from ciliated epithelial cells. This defense mechanism is coordinated with the actions of the airway lining fluid, rich in antioxidants, defensins, and lysozyme secreted by Clara cells and submucosal glands, along with mucous glycoproteins secreted by goblet cells (Figure 2) (66).

An exacerbated hRSV infection is characterized by several symptoms including severe chesty cough, wheezing, apnea and cyanosis and all of these symptoms can be signs of a lower respiratory tract infection (LRTI) (112). In infants, the leading pathology caused by LRTI is bronchiolitis, which has been described to involve an acute inflammation—mainly associated with exacerbated infiltration of neutrophils- necrosis of epithelial airway cells and increased production of mucus, among others (113, 114). Additionally, it has been described that the damage observed in the respiratory tract is not only induced by the viral infection itself, but also by the local production of cytokines (88). In the bronchioles samples from post-mortem patients, hRSV was detected mainly in the ciliated cells (115). Moreover, the majority of inflammation observed was at the submucosa level (115).

To understand the nature of this inflammation, the majority of the studies that have been performed in patients are focused on the analysis of the production of cytokines that coordinate the infiltration of immune cells. Since it is difficult to obtain bronchoalveolar lavage fluids (BALFs) samples from patients, these studies have been performed on ventilated hRSV-infected infants (116, 117). McNamara et al. collected these samples from term and pre-term infants to determine the inflammatory mediator profile in these children. An increase of the transcript and protein levels of cytokines such as IL-6, TNF-α, CXCL8, CCL3, CCL4, CCL2, and CCL5 was observed, as compared to control groups (Figure 2) (116, 117).

It has been reported that cytokines associated with a Th2-like response, such as IL-4, IL-6, IL-9, IL-10, and IL-13, are elevated in nasal washes and lungs of children with hRSV-induced LRTI (Figure 2) (72). Among these, IL-6 is a pro-inflammatory cytokine which has been described to play an essential role in the host immune response against hRSV infection (116). McNamara *et al*. also showed in pre-term and term infants with hRSV-induced bronchiolitis that IL-6 levels were elevated at day 1 of intubation, in term infants as compared to pre-term and control group (116). According to this observation, it is possible that IL-6 plays a relevant role in the hRSV pathogenesis in the lung of infected infants. When comparing the concentration of chemokines on the first day of intubation and the extubating day, no differences were found between these critical days (57). During another study performed in children under the age of 2 with clinical manifestations of respiratory obstruction and distress due to viral infection, different cytokines related to hRSV-infection were evaluated at three-time points: admission-hospitalization, discharge and 1 month after release (70). At the beginning of the study, the children admitted exhibited an increase in Th2-like cytokines such as IL-4, IL-5, and IL-13 (70). The increase in these cytokines decayed progressively until 1 month after discharge. In another study in children with signs of severe LRTI and positive for hRSV infection (71), cytokines were evaluated at two points: discharge and 1 year after release. Th2-like cytokines, such as IL-4 and IL-6, decayed 1 year after the infection. Surprisingly, IL-13 levels remained higher in the initially infected group when compared with the control group 1 year after the viral infection, although the authors could not rule out the effects of other diseases or environmental factors (71). Furthermore, children admitted in hospital with bronchiolitis due to hRSV-infection exhibited higher concentrations of IL-6 in nasal swabs as compared with their older siblings (118).

IL-10 has also been described as a key cytokine in the response against this virus (119–121). The varying levels and the role of IL-10 during hRSV infection have not been entirely determined, as IL-10 fluctuates with the age of children (120). Importantly, a study found that lower levels of IL-10 correlate with the severity of the hRSV disease in infants (120). Additionally, it has been described that in infants older than 3 months of age with mild hRSV infection exhibit high IL-10 levels, which can be related to a protector effect. Nonetheless, in infants below 3 months of age, high IL-10 levels were reported in those with severe bronchiolitis, therefore being considered as a hallmark of disease (121).

Interestingly, it has been reported that infants younger than 3 months, hospitalized with hRSV-induced bronchiolitis, presented elevated amounts of Th2-related cytokines in BALF samples, such as IL-3, IL-4, IL-10, and IL-13 (75). Furthermore, an increase of pro-inflammatory cytokines such as IL-1β, IL-6, TNF-α, and also IL-12-p40 -a Th1-like related cytokine- was also reported (75). Importantly, IL-3 -which is involved in the infiltration of immune cells that are related to the asthma development- and IL-12p40 are necessary for the secretion of IFN-γ. Therefore, the increase of both cytokines correlates with recurrent episodes of wheezing in hRSV infection (75).

IFN-γ is a cytokine that stimulates viral clearance by promoting anti-viral immune effector responses. Therefore, low levels of this cytokine in patients have been associated with a higher severity index in the bronchiolitis caused by hRSV (Figure 2) (122). Semple *et al*. reported that in infants hospitalized due to hRSV-induced bronchiolitis who needed oxygenation or ventilated support, IFN-γ levels in BALF were low when compared with the infants that never required oxygenation (122). These low IFN-γ levels correlated with increased severity of the disease and its reduction is significant in the development of the bronchiolitis (122). Contrary to these findings, recent studies performed by Thwaites *et al*. shows high levels of IFN-γ in patients from the pediatric intensive care unit (PICU) with hRSV-infection, along with high levels of IL-1 and IL-10 respect to the healthy controls (123). Also, reduced IFN-γ levels were detected in children with moderate bronchiolitis; however, in children with severe bronchiolitis, the levels of IL-17A and MUC5AC were increased (123). Considering the data mentioned above, the amount of IFN-γ in patients with hRSV-bronchiolitis is controversial.

Additionally, Semple et al. also analyzed the production of IL-9 (122). This cytokine is produced in Th9-like immune response and has been implicated in the severity of the hRSV pathology (124). The data obtained showed that IL-9 levels in BALF were increased in infants with severe bronchiolitis that required oxygenation. However, no differences were found when compared with infants that never needed oxygen supplementation (122). In another study performed in pre-term and term infants with hRSV bronchiolitis, the expression of the IL-9 mRNA in BALF was increased in both groups, as compared to control groups (124). Moreover, no significant differences were found in the levels of IL-9 transcript among pre-term and term infants. However, the protein secretion was increased in term infants when compared to both pre-term and control groups (124). Furthermore, the primary source of these cytokines in the lungs of hRSV-infected infants were neutrophils (124). Remarkably, it has been reported that IL-9 can upregulate genes involved in the mucus production in goblet cells, which could explain the elevated amounts of mucus in patients with hRSV-induced bronchiolitis (124). Additionally, it has been reported that IL-9 polymorphism has a different effect in the hRSV disease severity in boy and girls (125). The single nucleotide polymorphism (SNP) rs2069885 of the IL-9 gene was associated with higher susceptibility of severe disease caused by hRSV while in boys, is associated with a lower susceptibility (125).

Also, it has been described an association of the polymorphism of the IL-4 and IL-4Rα genes with hRSV disease severity (73). Hoebee *et al*. found that the−590T allele of the IL-4 gene was expressed more frequently in infants hospitalized by hRSV bronchiolitis compared to the control group (73). Moreover, the authors found an association of the hRSV disease severity and the IL-4 locus in children older than 6 months that were hospitalized by hRSV bronchiolitis (73). Additionally, this study found that 2 polymorphisms of the IL-4Rα gene, the I50V, and the Q551R. Only the Q551R SNP show an association with the children older than 6 months who were hospitalized by a severe hRSV bronchiolitis (73).

Another relevant cytokine reported upon hRSV infection is the thymic stromal lymphopoietin (TSLP), a cytokine associated with asthma development (126). Also, a strong association in Th2-like effector cytokines, such as IL-4 and IL-5, and IL-13 has been reported (127, 128). TSLP is secreted by epithelial cells associated with barriers (129) and bronchial smooth muscle cells (130, 131). In infants with hRSV-induced bronchiolitis, this cytokine was elevated as compared to healthy controls, suggesting that TSLP could play an essential role in the hRSV immunopathology (132).

Importantly, the response associated with IL-17 can be harmful to the patients, as mentioned above. Higher levels of IL-17 have been reported in patients with mechanic ventilation due to hRSV-induced bronchiolitis (76, 78). IFN-λ is a cytokine discovered in the year 2003 (77) and it has been reported to play a role in the establishment of the adaptive response to hRSV, with an increase in the secretion of IL-6, CXCL8, and IL-10 in peripheral blood mononuclear cells (PBMC) (133). Moreover, a deleterious effect of IFN-λ in hRSV infection has been seen (134), as a study of acute bronchiolitis-patients reported a significant increase in the transcription of IFN-λ in patients with increased respiration rate, a sign of acute bronchiolitis induced by hRSV-infection (134).

As we described earlier, chemokines are also involved in the inflammatory response elicited by hRSV-infection. One of these is CCL3, a small pleiotropic chemoattractant protein whose function is to attract or activate immune cells such as eosinophils, monocytes, basophils and lymphocyte subpopulations (135). This chemokine was increased in lower respiratory tract secretions from infants under 2 months old that were hospitalized with hRSV-induced bronchiolitis (135). Interestingly, this increase was correlated with the detection of eosinophil degranulation products, which suggests that CCL3 has an active role in this process during hRSV-induced bronchiolitis (135). In addition to this, it was also reported that CCL5 was increased in these infants (135). CCL5 is a chemoattractant cytokine that principally recruits monocytes, T cells, and eosinophils, acting via three chemokine receptors: CCR1, CCR3, and CCR5 (136). Evidence obtained from children with hRSV infection shows an increase of the CCL5 protein levels in both upper and lower airway secretions, and levels of CCL5 in upper airway secretions correlate positively with disease severity (137, 138). Recently a prospective study of 173 patients with bronchiolitis caused by hRSV was performed, holding 536 healthy controls whose samples of nasopharyngeal aspirate were taken (139). Therein, the authors found a single SNP in CCL5 (rs2107538^*^CT), exhibiting an association with hRSV-bronchiolitis and also with the need for mechanical ventilation (139). These data suggest that CCL5 contributes to bronchiolitis leading to airways damage in patients.

Furthermore, McNamara et al. also evaluated this chemokine in BALF from infants that required ventilation support and found an increase at the first day of the mechanical ventilation, but these levels decreased over time (117). This phenomenon was also observed for CXCL8 (117), which is a chemokine that attracts mainly neutrophils, one of the most frequent immune cells found in the airways of hRSV-infected infants (140). Subsequently to these results, another study performed in BALF samples from intubated infants reported elevated levels of CXCL8 transcript, which also correlates with the finding of this chemokine in nasopharyngeal aspirates (NPA) (141). Thus, the NPA samples might be an excellent alternative to study the implications of the infection caused by hRSV in the respiratory tract (Figure 2).

## Cytokines Secreted by Epithelial Cells in Response to the hRSV Infection *in Vitro*

The majority of the knowledge available about the induction of pro-inflammatory cytokines and chemokines production upon hRSV infection has been described *in vitro* using airway epithelial cells (AECs) models such as A549, primary human small airway epithelial cells (SAECs), BEAS-2B and primary normal human bronchial epithelial cells (NHBE), among others (88, 142, 143). The data obtained using these models can vary depending on the cell line. According to this, experiments in the A549 cell line (human alveolar type II-like epithelial) with the Long strain of hRSV showed that infection with hRSV induces the secretion of IL-6, CCL3, and CCL5 at 48 h post-infection as compared to non-infected cells (144, 145). On the other hand, a study performed in this cell line but with a different strain and subgroup of hRSV obtained from clinical isolates showed that the induction of IL-6 and CCL5 could be variable and dependent on the virus strain used (146).

BEAS-2B is an SV40 transformed human normal bronchial epithelium cell line that exhibits a limited susceptibility to hRSV-infection and profile of virus resistance as compared to the A549 cell line (142). Infection of this cell line with the hRSV Long strain showed an increase in the transcript levels for CXCL8 at 4 h post-infection, which was observed up to 24 h post-infection (143). Regarding the upregulation of IL-6, it was observed only at 96 h post-infection (143). However, another study performed using the hRSV Long strain showed that CXCL8 levels were not changed upon infection with hRSV, while CCL3 and CCL5 levels were increased (64). Importantly, the authors observed that the amounts of CCL5 produced by the epithelial cells were enough to attract eosinophils (64). Furthermore, infected BEAS-2B cells with the hRSV strain A2 also exhibited an induction in the secretion of IL-6 and CXCL8 as compared to non-infected cells (145).

Currently, primary or normal epithelial cells are the most used model for hRSV infection as it is thought to be representative of the effects of hRSV infection in the respiratory tract. Accordingly, it has been reported that primary AECs obtained from hRSV-infected infants exhibited higher viral titers as compared to the BEAS-2B cell line when infected with the same virus (147). Besides, the amounts of IL-6 and CXCL8 were higher in the primary AECs as compared to BEAS-2B cells (147). Considering these data, AECs can be considered as an excellent model for understanding the effects of hRSV-infection and the production of cytokines and chemokines as may occur in infants.

Additionally, in a study *in vitro* using a WT hRSV A2 strain (6340WT) and a recombinant strain that lacks the G-protein gene (6340ΔG), infection of NHBEs cells induced the secretion of CCL2, CCL5, and CXCL8 by both viruses (148). However, only the recombinant virus was able to promote the secretion of CXCL10 in NHBEs (148). On the other hand, both F- and G-protein promoted the secretion of CXCL8 and CXCL10, whereas only G-protein induced the secretion CCL5 (148). In contrast to these findings, infection with the hRSV Long strain in NHBEs cells did not lead to the secretion of CCL2 and CCL3, but the levels of CCL5 were increased as compared to uninfected cells (149). Additionally, it has been described that hRSV infection in NHBEs cells induced the expression of TSLP transcript at 12 h post-infection and TSLP secretion exhibited a peak at 24 h post-infection as compared to ultraviolet (UV)-hRSV inactivated (150). This phenomenon was also seen in NHBEs cells obtained from asthmatic patients and infected with hRSV, as TSLP concentration were high when compared to healthy patients with hRSV-infection (150). Moreover, studies using A549 cells co-transfected with the human TSLP promoter with a reporter, and a dominant-negative form of RIG-I (DN-RIG-I), showed that hRSV-infection could induce activation of this pathway to increase TSLP expression (150).

## hRSV Induced Cytokine Production and Tissue Damage in Mice

It has been described that hRSV-infected BALB/c mice can exhibit increased levels of IL-6 in BALFs at 12 h post-infection that remains elevated up until 14 days post-infection (59). Similar results were observed in lungs parenchyma and sera of hRSV-infected mice (59). The contribution of IL-6 to the hRSV immunopathology was evaluated by the depletion of this cytokine one day before hRSV infection, parameters of disease, such as weight loss were more severe (59). Furthermore, in these hRSV-infected mice the lung vascular permeability was evaluated by measurement of albumin in the airways, which was increased as compared to the isotype control at 7, 11, and 14 days post-infection (59). Further, in the absence of IL-6, hRSV-infected mice displayed an increase of lymphocyte recruitment at 7 days post-infection, while neutrophil infiltration was similar to the isotype control (59). These results suggest that the early production of IL-6 is essential to control the severity of the disease and to limit lung damage.

Furthermore, it has also been described that hRSV infection promotes an increase of IL-1β, TNF-α, IFN-γ IL-12, IL-6, CCL3, and CCL5 in BALF samples from mice (151). The elevated levels of IL-1β and TNF-α on the first day of hRSV infection correlate with the peak of weight loss, whereas increased levels of IL-12 were found before the induction of IFN-γ (151). Besides, histological analyses have shown that hRSV infection produces changes in the lung that are associated with airway and vascular cuffing and interstitial pneumonia (144). On the other hand, an effect of TNF-α alone over the hRSV-infection has not been demonstrated with knockout mice. However, in a study in BALB/cJ mice with pretreatment with antibody for TNF-α before the hRSV-infection, mice showed a significant increase of weight loss and slow recovery as compared to control mice (152). Therefore, these observations suggest that TNF-α can be established as a participant in the hRSV-infection, and in the absence of this cytokine the mice showed a delay in the viral clearance.

The role of IFN-γ during hRSV pathogenesis was evaluated using both an IFN-γ knockout mice model and the blockade of IFN-γ (153). The data obtained from this study shows that, both in IFN-γ knockout (IFN-γ^−/−^) mice and in the anti-IFN-γ treated mice, the immune cell infiltration (principally neutrophils and eosinophils) in BALF samples were higher than in control mice. However, when the respiratory rate was evaluated [the ratio between inspiration time and expiration time (Ti/Te)] the anti-IFN-γ treated hRSV-infected mice shows no difference in the ratio compared to control mice (153). Besides, in the absence of IFN-γ also increase the viral load of these mice compared to control mice. These results suggest that IFN-γ plays a dual role during hRSV infection, been necessary to control the viral replication and also prevents the obstruction of the airways (153).

Regarding to the role of chemokines, has been reported that elevated concentrations of CCL3 and CCL5 at day one post-infection are consistent with recruitment of monocytes and lymphocytes into the mice lungs (151, 154). Additionally, it has been described that CCL5 induction by hRSV infection contributes to a subsequent allergic pulmonary inflammation (155). Moreover, in mice, the secretion of CCL5 in the lungs was correlated with airway hyperreactivity (AHR). This association was evaluated by antibody neutralization of CCL5, showing that while viral loads were not affected by this treatment, a significant decrease for hRSV-induced AHR was observed, down to control mice levels (156). Furthermore, it was described that CCL5 exhibits a biphasic response during the hRSV infection, with an initial phase of innate immune response and a second phase consisting of lymphocyte-mediated responses (157). Besides, mice sensitized with recombinant vaccinia expressing G-hRSV protein (rVV-G) showed a significant increase of both mRNA and protein levels for CCL5 during the first 24 h post-infection (157). Then, CCL5 is also increased in the second phase of hRSV infection at 168 h post-infection (157). To understand the role of CCL5 when viral replication was eliminated, an inhibitory analog of CCL5, Met-RANTES, was used to treat hRSV-infected mice. These studies showed that mice treated with Met-RANTES exhibited a significant reduction of CD4^+^ and CD8^+^ T cell recruitment into the lungs after infection (157). Along these lines, blockage of CCL5 reduced both weight loss and eosinophilia, suggesting that this cytokine plays an essential role during lung inflammation (157). Accordingly, the induction of CCL5 by hRSV infection is involved in lung inflammation, although there is no evidence of a contribution or a direct role in airway damage.

Regarding the contribution of CCL3 to hRSV infection, it was shown that equivalent to CCL5, CCL3 displays a biphasic expression both for mRNA and protein, at day 1 and 7 post-infection (158). Moreover, blockage of CCL3 with a neutralizing antibody showed no change in the recruitment of NK cells and did not affect viral loads in the lungs of hRSV-infected mice after 4 days of infection (158). However, at 7 days post-infection, the number of CD4^+^ and CD8^+^ T cells was reduced in the lungs of infected mice (158). Accordingly, hRSV-infected BALB/c WT mice exhibited an infiltration of about 80% of mononuclear cells close to vessels and bronchioles, while CCL3^−/−^ hRSV-infected mice exhibited a decrease of infiltrating cells in the lungs. Interestingly, in both mice, the viral loads were equivalent (154). Additionally, in CCL3^−/−^ hRSV-infected mice the mRNA of CCL5, CCL2, and CXCL2 were decreased as compared to their wildtype littermates, suggesting that CCL3 is required for the development of the hRSV-induced immunopathology (154). Despite these data, there is no direct evidence of the pulmonary damage caused by CCL3, which be relevant to determine.

Related with the production of TSLP in hRSV-infected mice, it has been reported that at the peak of the immunopathology, high amounts of this cytokine are produced (150). The contribution of TSLP to the hRSV pulmonary immunopathology was analyzed using TSLP KO mice and results showed that expression of Gob5, IL-13, and mucus production decreased as compared to hRSV-infected WT mice (150). Moreover, Stier et al. showed that knockout mice for the TLSP receptor (TSLPR KO) infected with hRSV displayed moderate mucous cell metaplasia, as the WT hRSV-infected mice. However, the accumulation of intraluminal mucus was lower when compared to WT mice (159). The airways obstruction of both hRSV-infected WT and TSLPR KO mice, was evaluated by methacholine challenge. Consistently, hRSV-infected WT mice displayed an increase in the airway reactivity (increased amounts of methacholine) as compared to the hRSV-infected TSLPR KO mice. These later animals showed only minor symptoms of the disease, which were equivalent to the mock-treated mice (159). Accordingly, these results suggest that TSLP activity is relevant for the hRSV immunopathology and that also contributes to lung damage in murine models (150).

As described above, most of the work in this field suggests what cytokines are either up- or down-modulated during hRSV infection. However, little or nothing has been reported about the direct contribution of these mediators to the airway damage caused by hRSV. The development of new methodological approaches is still necessary to achieve a better understanding of the effects that this virus produces on the respiratory tract by inducing inflammatory mediators. However, it could be possible to suggest that, like what is seen for the upper respiratory tract, hRSV exhibits several redundant mechanisms that induce damage and inflammation in the lower respiratory tract.

## hRSV Infection is Associated With Elevated Levels of Cytokines in the CNS

As described above, hRSV infection induces cytokines that damage the respiratory tract, but also these cytokines could affect the CNS. Years ago, a small number of hRSV-infected patients were reported to exhibit clinical signs associated with neurological complications, such as seizures (160, 161), apnea (12), encephalopathy (162) and encephalitis (163, 164). Nowadays, the cases of neurological abnormalities related to hRSV infection reported are increasing. However, our knowledge regarding the mechanisms involved in this phenomenon remains limited and controversial (Figure 1).

One of the first findings in patients with neurological manifestations associated with hRSV infection was the detection of virus-specific antibodies in cerebrospinal fluid (CSF) (165). Later, after many efforts to find viral genetic material in CSF, hRSV RNA belonging to the serogroup B was detected in the CSF of an infant with febrile convulsion and pneumonia (13). Researchers not only have focused on hRSV detection, but also on the possible production of cytokines that could be a consequence of viral infection and that could explain the symptoms affecting the CNS. Accordingly, an increase of IL-6 in CSF from an hRSV-infected patient was reported (14). The observation that serum IL-6 levels in these patients were normal (14) would suggest that this cytokine is produced locally in the CNS, most likely by CNS-resident cells, such as microglia and astrocytes. A report about 3 clinical cases where children infected by hRSV suffered from seizures, showed that the levels of IL-6 were increased and that the serogroup of hRSV found in the CFS belonged to the serogroup A (166). Additionally, the same authors also found viral RNA in the CSF of a different cohort of hRSV-infected patients, with increased levels of IL-6, IL-8, CCL2, and CCL4, suggesting that these inflammatory mediators may play a critical role during the hRSV-infection in the CNS pathogenesis (15). Importantly, the increased levels of IL-6 correlate with the severity of the CNS encephalitis mediated by a cytokine storm, which can be useful as a molecular marker of neurological prognosis (167). Based on all the data described above, it is possible that hRSV spreads from the lungs to the CNS and infects local cells, initiating an inflammatory immune response mediated by cytokines.

As we mentioned above, there is controversy in this field due to reports in which hRSV-derived genetic material was not found in CSF samples from patients with severe bronchiolitis (168). Analyses of blood and CSF samples from 10 patients with apneas showed that only 7 were positive for hRSV (168). This study showed that hRSV RNA was detected in PBMC of two patients, but was not found in their CSF (168). Possible explanations for this controversy could be due to differences in the clinical signs of the patients, to the hRSV serogroups found infecting them and also to technical differences used by the researchers.

Although there is clinical relevance in the CNS pathologies caused by hRSV infection, there is little research in this aspect that could provide conclusive evidence. A study using the mouse model described that hRSV could infect sensory neurons in the lungs through the interaction of the G-hRSV glycoprotein with the chemokine receptor CX3CR1 located at the surface of these cells (169). Experiments with mouse neuronal primary cultures showed that hRSV infected about 5% of these cells and this percentage decrease when CX3CR1 was blocked (169). Nevertheless, in this study, the authors did not evaluate the effect of hRSV infection in these cells or whether the neurons secreted inflammatory mediators. To approach these questions, neuronal N2a cells were infected with hRSV showing that these cells secrete IL-6 and TNF-α *in vitro* (170).

While these reports advance the knowledge in this field, there is still no evidence of neuronal infection by hRSV *in vivo*. In this regard, Espinoza *et al*. described that hRSV could be detected in several areas of the brain from infected mice, such as the cortex, ventromedial hypothalamic nucleus, and hippocampus (171). Interestingly, the finding of the virus in the hippocampus led to hypothesize that behavioral and learning processes may be altered. Marble burying (MB) and Morris Water Maze (MWM) test were performed 30 days after hRSV infection to test this hypothesis. In both trials, behavioral (MB) and spatial learning (MWM), performance was altered in hRSV-infected mice (171). The authors also evaluated the possible impairment in the functionality of the synaptic plasticity in the hippocampus. The data shows that the long-term potentiation (LTP) and the long-term depression (LTD) were altered in hRSV-infected mice, suggesting damage in the brain of these animals (171). It is possible to think that impairment in the behavior and learning is due to the neuronal infection by hRSV, which alters the normal function of these cells. Besides, it is also possible that hRSV infection promotes the secretion of several cytokines in the CNS, either by neurons or other resident cells, which could contribute to this neurological-associated phenomenon. However, more research is still necessary in this field to further advance our knowledge of the effects that this virus has on our CNS.

## Concluding Remarks

HRSV remains one of the primary viral agents causing respiratory tract infections worldwide, for which there is no vaccine available. Once hRSV infection reaches the epithelium of the respiratory tract, it produces several symptoms such as wheezing, apnea, cyanosis, and bronchiolitis, related to acute lower tract infection. Most of the damage seen in patients with complications associated with hRSV infection is caused by an exacerbated immune response triggered mainly by the cytokines secreted by the infected cells of the respiratory tract epithelium.

In human studies, cytokines and chemokines have been detected in nasopharyngeal aspirates, tracheobronchial aspirates or bronchoalveolar lavage fluids, in children with mechanic ventilation due to bronchiolitis associated with hRSV infection. In these patients -usually children younger than 2 years of age- the cytokines that predominated were IL-4, IL-5, IL-6, IL-10, and IL-13. A low concentration of cytokines associated with a Th1-like response such as IFN-γ is also seen, which could be considered as a severity index. Also, chemokines such as CCL3, CCL5, and CXCL8 are increased in the lower respiratory tract of individuals infected with hRSV. These components contribute to generating a severe pathology in the patients, which is associated with an unbalance between Th1- and Th2-like cytokines, and an increase in chemokines that attract more inflammatory cells like granulocytes, which in turn generates a deleterious effect on the patient. Moreover, the secretion of many of the cytokines described above has also been seen in mice models, with associated tissue damage, although further studies are still required to fully elicit the specific role of each cytokine in this pathology.

Furthermore, infection by hRSV seems to reach CNS, which produces high levels of IL-6 in the zone. This infection might generate problems in the behavior and learning process of the children, but further studies are required to elucidate more information in this regard.

## Author Contributions

All authors listed have made a substantial, direct and intellectual contribution to the work, and approved it for publication.

### Conflict of Interest Statement

The authors declare that the research was conducted in the absence of any commercial or financial relationships that could be construed as a potential conflict of interest.
